# Children with single ventricle heart disease have a greater increase in sRAGE after cardiopulmonary bypass

**DOI:** 10.1177/02676591231189357

**Published:** 2023-07-19

**Authors:** Bonnie A Brooks, Pranava Sinha, Steven J Staffa, Marni B Jacobs, Robert J Freishtat, Jason T Patregnani

**Affiliations:** 1 Division of Pediatric Critical Care Medicine, Mattel Children’s Hospital, 8783 University of California Los Angeles, Los Angeles, CA, USA; 2 Division of Critical Care Medicine, Children’s National Hospital, Washington, DC, USA; 3 Department of Pediatric Cardiac Surgery, 14400 M Health Fairview University of Minnesota, Minneapolis MN, USA; 4 Division of Cardiovascular Surgery, Children’s National Hospital, George Washington University School of Medicine and Health Sciences, Washington, DC, USA; 5 Department of Anesthesiology, Critical Care and Pain Medicine, Harvard University, 385266 Boston Children’s Hospital, Boston, MA, USA; 6 Department of Obstetrics, Gynecology, and Reproductive Sciences, 8784 University of California, San Diego, CA, USA; 7 Division of Biostatistics and Study Methodology, Children’s National Hospital, Washington, DC, USA; 8 Center for Genetic Medicine Research, Children’s National Hospital, Washington, DC, USA; 9 Departments of Pediatrics, Emergency Medicine, and Genomics & Precision Medicine, George Washington University School of Medicine and Health Sciences, Washington, DC, USA; 10 Division of Pediatric Critical Care Medicine, Maine Medical Center, Tufts University School of Medicine, 23365 Barbara Bush Children’s Hospital, Portland, ME, USA; 11 Division of Pediatric Cardiac Critical Care, Children’s National Hospital, George Washington University School of Medicine, Washington, DC, USA

**Keywords:** cardiopulmonary bypass, congenital heart disease, single ventricle, biomarker, inflammation, acute lung injury, acute respiratory distress syndrome

## Abstract

**Introduction:**

Reducing cardiopulmonary bypass (CPB) induced inflammatory injury is a potentially important strategy for children undergoing multiple operations for single ventricle palliation. We sought to characterize the soluble receptor for advanced glycation end products (sRAGE), a protein involved in acute lung injury and inflammation, in pediatric patients with congenital heart disease and hypothesized that patients undergoing single ventricle palliation would have higher levels of sRAGE following bypass than those with biventricular physiologies.

**Methods:**

This was a prospective, observational study of children undergoing CPB. Plasma samples were obtained before and after bypass. sRAGE levels were measured and compared between those with biventricular and single ventricle heart disease using descriptive statistics and multivariate analysis for risk factors for lung injury.

**Results:**

sRAGE levels were measured in 40 patients: 19 with biventricular and 21 with single ventricle heart disease. Children undergoing single ventricle palliation had a higher factor and percent increase in sRAGE levels when compared to patients with biventricular circulations (4.6 vs. 2.4, *p* = 0.002) and (364% vs. 181%, *p* = 0.014). The factor increase in sRAGE inversely correlated with the patient’s preoperative oxygen saturation (Pearson correlation (r) = −0.43, *p* = 0.005) and was positively associated with red blood cell transfusion (coefficient = 0.011; 95% CI: 0.004, 0.017; *p* = 0.001).

**Conclusions:**

Children with single ventricle physiology have greater increase in sRAGE following CPB as compared to children undergoing biventricular repair. Larger studies delineating the role of sRAGE in children undergoing single ventricle palliation may be beneficial in understanding how to prevent complications in this high-risk population.

## Introduction

Despite many improvements in the perioperative and postoperative management of children with congenital heart disease (CHD), children with single ventricle heart disease (SVD) continue to be at risk for significant postoperative sequelae as compared to children undergoing repair of biventricular heart disease (BVD).^1,2^ Strategies to minimize extracardiac injury in the perioperative period may be important for both acute and long-term successes in the patient undergoing single ventricle palliation.

Staged palliation for SVD requires at least two to three exposures to cardiopulmonary bypass (CPB) in the first 5 years of life. Though essential, CPB has significant systemic effects and can lead to end organ injury.^3–7^ Pediatric patients are thought to be at highest risk for complications on account of the smaller patient size-to-circuit ratios, an exaggerated inflammatory response, and an increased susceptibility of pediatric end organs to reperfusion injury.^3–7^ In addition to unique cardiac anatomies, patients with single ventricle physiology present other considerations such as baseline cyanosis and differences in volume loading conditions. How these characteristics alter the postoperative inflammatory response to CPB and outcomes in CHD is an ongoing area of research.^8,9^

Biomarker research may help clinicians predict which patients are more likely to experience low cardiac output syndrome, postoperative lung injury, and other acute complications.^6,10–12^ One biomarker the receptor for advanced glycation end products (RAGE) is a transmembrane protein predominantly found on type 1 alveolar cells in the lung.^13,14^ When ligand bound, RAGE incites a pro-inflammatory cascade, notably via the nuclear factor kappa light chain enhancer of activated B cells (NF-kB) pathway.^13–15^

RAGE undergoes proteolytic cleavage and alternative splicing events producing different isoforms, including soluble RAGE (sRAGE).^16,17^ sRAGE has been investigated as an early and sensitive biomarker of lung injury in many different pathologic states including acute respiratory distress syndrome (ARDS), chronic obstructive pulmonary disease, pediatric asthma, and bronchiolitis.^18–21^ sRAGE has also been evaluated for its utility as a biomarker in assessing postoperative lung injury and dysfunction after cardiac surgery.^11,22,23^ Adult patients undergoing CPB have elevated levels of postoperative sRAGE compared to patients undergoing off-pump cardiac surgeries,^11,22^ and higher levels are associated with worse postoperative pulmonary function and intensive care unit (ICU) length of stay.^11^ Children undergoing repair of acyanotic congenital heart disease requiring CPB have been shown to have an acute increase in postoperative sRAGE levels. These elevated levels are positively associated with severity of acute lung injury, length of time on mechanical ventilation, and hospital length of stay.^23^ Recently, a prospective, multi-institutional study of children with ARDS found a positive association between sRAGE levels and extrapulmonary organ dysfunction and mortality,^24^ implicating a broader scope for sRAGE not limited to lung epithelial damage. These potentially prognostic qualities of sRAGE led us to consider whether this protein could be utilized in our population of patients with CHD undergoing CPB. We specifically sought to compare sRAGE levels in children with both single ventricle and biventricular physiologies and hypothesized that patients undergoing single ventricle palliation would have increased levels of sRAGE after CPB as compared to those undergoing repair for BVD.

## Methods

### Patient population and sample collection

All children under the age of 5 years undergoing surgical correction for congenital heart defects at Children’s National Hospital from August 2017 until March 2018 were eligible for this study. Patients who required postcardiotomy extracorporeal membrane oxygenation were excluded from the study. This study was approved by the Institutional Review Board at Children’s National Hospital (Study ID: Pro00008867) and written informed consent for participation was obtained from all families. Following induction of anesthesia, 2 mL of blood was collected via an indwelling arterial catheter into an EDTA tube. Another 2 mL blood sample was collected after separation from bypass, 15 min following protamine administration. Serum samples were centrifuged immediately for 20 min at 24°C at a speed of 800 revolutions per minute. Plasma was then separated and stored at −80°C until further processing. Demographic and clinical data were collected from the electronic medical record.

Modalities of extracorporeal circulation, the CPB set up, anesthetic technique, and transfusion management were largely standardized for all patients, and there was no variation in oxygenation or pH management between groups. Pump prime consisted of Plasma-Lyte A (Baxter, Deerfield, IL), with the addition of packed red blood cells (RBC) and fresh frozen plasma (FFP) to achieve a target hematocrit above 28%. A hemoconcentrator was used whenever extra volume was present. Once the patient was successfully cannulated, CPB was managed with a pH-stat strategy and 100% FiO_2_. Patients were cooled to a temperature determined by the operating surgeon. During cooling, perfusion flow rates were maintained at a cardiac index of at least 2.4 L/min/m^2^. Deep hypothermic circulatory arrest or low flow hypothermic bypass was utilized based upon the procedure needed. As rewarming phase was initiated, hemoconcentration was performed to remove additional volume. At the resolution of CPB, blood products, including RBC, platelets, and cryoprecipitate were administered to control bleeding and to maintain a hematocrit above 28%.

### Determination of sRAGE in plasma

Total sRAGE was measured using an enzyme-linked electrochemiluminescence assay provided by MesoScale Discovery® (Gaithersburg, MD, USA) following the manufacturer’s provided protocol. Human RAGE antibody was provided by R&D Systems® (Minneapolis, MN, USA).

### Statistical analysis

Demographic and clinical characteristics were compared between children with SVD and those with BVD using Wilcoxon-Mann-Whitney or Fisher’s exact tests, as appropriate. Absolute changes in sRAGE levels were compared using Wilcoxon signed-rank test. Associations between preoperative peripheral oxygen saturation (SpO_2_) and factor or percentage changes in sRAGE levels were performed using linear regression modeling and the Pearson correlation coefficient. Multivariable linear regression analysis was used to determine independent associations between variables and percentage and factors changes in sRAGE levels. Multivariable analyses were used to control for patient age at the time of study given a lack of baseline range of sRAGE levels in the pediatric population and the age differences between the SVD and BVD groups. Results from multivariable modeling are presented as adjusted coefficients, 95% confidence intervals, and Wald *p* values. Stata 15.0 (StataCorp, College Station, Texas) and SAS 9.4 was used for statistical analysis. A two-tailed alpha level of 0.05 was used to determine statistical significance.

## Results

### Demographics

Forty-one children who underwent CPB for repair of CHD were enrolled in this study with one patient excluded for failure to separate from CPB and requiring transition to extracorporeal membrane oxygenation support. Children with SVD were older [median 207 (IQR:172, 770) vs. 112 (41, 393) days, (*p* = 0.01)] and weighed more at the time of surgery [median 8.8 (IQR: 6.7, 12.4) vs. 5.2 (IQR: 4.0, 7.3) kg, (*p* = 0.004)]. Children with SVD had more prior bypass operations (*p* < 0.001). There were no differences in any other demographic feature (Table 1). A summary of the patient’s cardiac anatomy and the surgeries that were performed are summarized in Table 2.

**Table 1.** Demographics based on single versus biventricular heart disease.

	Single ventricle (*N* = 21, 52.5%)	Biventricular (*N* = 19, 47.5%)	*p*-value
Sex (*n*, %)			0.44
Male	17 (81.0)	14 (73.7)	
Female	4 (19.0)	5 (26.3)	
Age at surgery (days) (median, IQR)	**207 (172, 770)**	**112 (41, 393)**	0.01*
Weight at surgery (kg) (median, IQR)	**8.8 (6.7, 12.4)**	**5.2 (4.0, 7.3)**	0.004*
Race/Ethnicity (*n*, %)			0.66
Black	9 (42.9)	7 (36.8)	
White	8 (38.1)	7 (36.8)	
Hispanic	3 (14.3)	2 (10.5)	
Asian/other	1 (4.8)	3 (15.8)	
Premature birth (*n*, %)			1.00
No	19 (90.5)	18 (94.7)	
Yes	2 (9.5)	1 (5.3)	
Preoperative saturation (SpO_2_ %) (median, IQR)	**84 (79, 88)**	**98 (97, 100)**	< 0.001*
Previous CPB operation (*n*, %)			< 0.001*
No	**3 (14.3)**	**18 (94.7)**	
Yes	**18 (85.7)**	**1 (5.3)**	

Data are presented as number of patients (%) or median interquartile range (IQR) as appropriate. *p*-value based on Wilcoxon-Mann-Whitney for continuous variables and Fisher’s exact test for categorical variables * = statistical significance.

kg = kilograms.

**Table 2.** Cardiac anatomy and type of surgery.

Single ventricle lesion	Number (%)	Single ventricle surgery	Number (%)
DORV variant	8 (38)	Bidirectional Glenn/Superior	11 (52)
HLHS	5 (24)	Cavopulmonary anastomosis	
DILV	3 (14)	Fontan completion	9 (43)
PA/IVS	2 (10)	Norwood/mBTT shunt	1 (5)
Tricuspid atresia	2 (10)		
Other	1 (5)		

Biventricular lesion	Number (%)		
VSD	5 (26)		
TOF	4 (21)		
CAVC	2 (11)		
DORV variant	2 (11)		
d-TGA	1 (5)		
Truncus arteriosus	1 (5)		
ASD	1 (5)		
Other	3 (16)		

Data are presented as counts (%).

ASD = atrial septal defect; CAVC = complete atrioventricular canal; DILV = double-inlet left ventricle; DORV = double-outlet right ventricle; HLHS = hypoplastic left heart syndrome; mBTT = modified Blalock-Thomas-Taussig; PA = pulmonary artery; PA/IVS = pulmonary atresia with intact ventricular septum; d-TGA = d-transposition of the great arteries; TOF = tetralogy of Fallot; VSD = ventricular septal defect.

### Clinical data

There were no differences in duration of CPB, cross-clamp, or deep hypothermic circulatory arrest between our two populations (Table 3). Patients with SVD received more packed RBC intraoperatively [median 160 (IQR:130, 197) vs. 110 (50, 140) mL, (*p* = 0.02)] but received similar volumes of other blood products. SVD patients also had higher volumes of 24-hour postoperative chest tube output [39 (29, 74) vs. 24 (12, 37) mL/kg], (*p* = 0.007)] (Table 3). There were no differences in days of mechanical ventilation or hospital length of stay.

**Table 3.** Intraoperative and postoperative clinical data by ventricle status.

Clinical characteristic	Single ventricle (*N* = 21, 52.5%)	Biventricular (*N* = 19, 47.5%)	*p*-value
CPB time (min)	91.5 (83.0, 100.0)	74.0 (60.0, 110.0)	0.29
Crossclamp time (min)	31.0 (0, 91.5)	39.0 (22.0, 63.0)	0.53
Deep hypothermic circulatory arrest time (min)	0 (0, 5)	0 (0, 0)	0.41
PRBC administered (mL)	**160 (130, 194)**	**110 (50, 140)**	0.02*
Platelets administered (mL)	108 (87, 126)	114 (70, 131)	0.91
Cryoprecipitate administered (mL)	30 (20, 30)	20 (20, 30)	1.00
FFP administered (mL)	30 (0, 60)	0 (0, 122)	0.43
Chest tube output (mL/kg)	**39.2 (28.6, 73.5)**	**23.5 (12.4, 36.8)**	0.007*
Mechanical ventilation (days)	0 (0, 3)	1 (0, 3)	0.72
Hospital length of stay (days)	7 (6, 17)	6 (4, 11)	0.13
sRAGE at baseline (pg/mL)	**628.5 (349.9, 1167.1)**	**1722.2 (883.7, 2216.5)**	**0.02**
sRAGE after bypass (pg/mL)	3032.3 (1244.6, 7095.6)	3959 (1840.4, 5455.4)	0.285

Data are presented as number of patients (%) or median interquartile range (IQR). * = statistical significance. *p*-value based on Wilcoxon-Mann-Whitney for continuous variables and Fisher’s exact test for categorical variables. CPB = cardiopulmonary bypass; kg = kilogram; min = minutes; mL= milliliters; pg = picograms; PRBC = packed red blood cells.

### sRAGE concentrations

**Figure 1.**
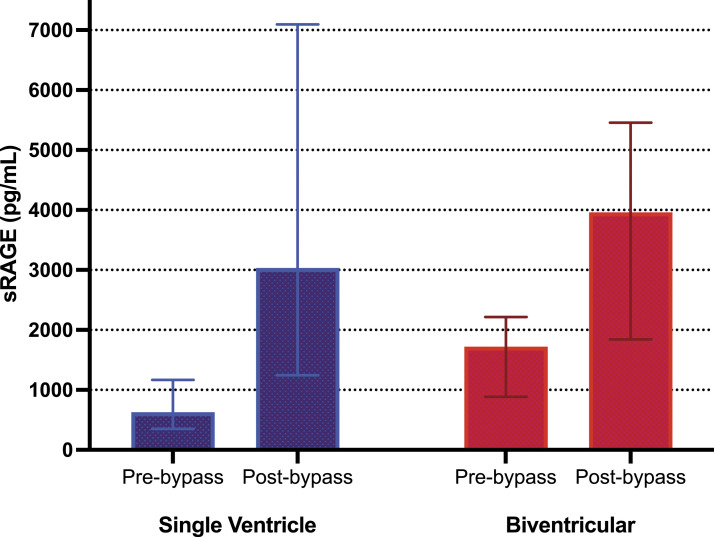
Soluble RAGE (sRAGE) levels in plasma before and immediately after CPB by ventricle status. A greater factor increase was noted in single ventricle patients (*p* = 0.002) and a greater percent change was noted in single ventricle patients (*p* = 0.014). Data are presented as median and the interquartile range.

**Figure 2.**
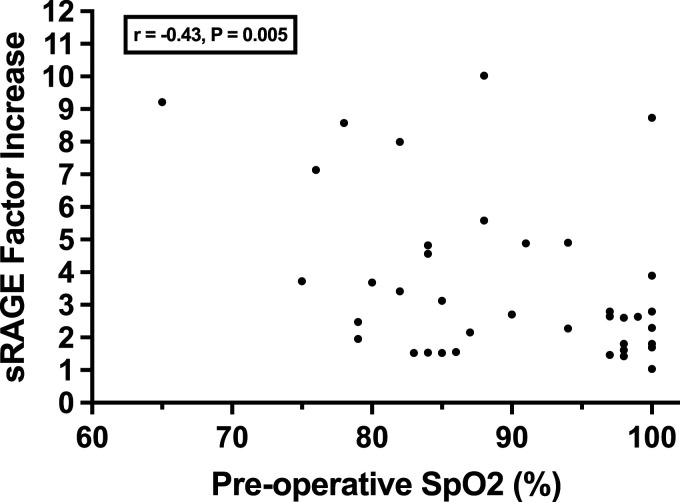
Pearson correlation between factor increase in sRAGE based on patient preoperative oxygen saturation. \ Points are plotted by sRAGE factor increase and baseline oxygen saturation. A moderate inverse relationship was found between these two variables.

All patients in the study had higher concentrations of sRAGE post-bypass than pre-bypass with a median absolute change of 2113 pg/mL, representing a factor increase of 2.8 times (*p* < 0.0001). Children with SVD had a higher factor increase and mean percent increase in sRAGE compared to those with biventricular circulation (4.6 vs. 2.4 factor increase, *p* = 0.002), (364% vs. 181%, *p* = 0.014) controlling for patient age at the time of surgery (Figure 1). Patients with SVD had lower levels of sRAGE prior to bypass when compared to those with biventricular defects [median 629 (IQR: 350,1167) vs. 1722 (884, 2217) pg/mL, (*p* = 0.02)] (Table 3). There was no difference in postoperative sRAGE levels between the two groups (*p* = 0.285, Table 3). Both factor and percent increase in sRAGE inversely correlated with the patient’s preoperative SpO_2_ (Pearson correlation (r) = −0.43; *p* = 0.005; (r) = −0.38; *p* = 0.014) respectively (Figure 2). On multivariable linear regression analyses, we found significant positive independent associations for sRAGE factor and percent increase with packed RBCs administered intraoperatively (coefficient = 0.011; 95% CI: 0.004, 0.017; *p* = 0.001) and (coefficient = 0.969; 95% CI: 0.333, 1.606; *p* = 0.004), respectively (Table 4).

**Table 4.** Multivariate analysis of sRAGE factor and percent increase.

Multivariable analysis of sRAGE factor increase			
Variable	Adjusted coefficient	95% CI	*p*-value
Age	0	(−0.002, 0.002)	0.948
CPB time	−0.009	(−0.027, 0.007)	0.239
**Intraoperative PRBC**	**0.011**	**(0.004, 0.017)**	**0.001** ^a^
Prematurity	0.309	(−2.447, 3.064)	0.821

Multivariable analysis of sRAGE percent increase			
Variable	Adjusted coefficient	95% CI	*p*-value
Age	−0.008	(−0.189, 0.173)	0.927
CPB Time	−0.836	(−2.518, 0.845)	0.320
**Intraoperative PRBC**	0.969	**(0.333, 1.606)**	**0.004** ^a^
Prematurity	−19.98	(−297.19, 257.22)	0.884

Multivariable modeling was done using multivariable linear regression analysis.

^a^Statistically significant.

## Discussion

In this study, we compared sRAGE levels in children with SVD and BVD undergoing CPB. Children with SVD started with lower baseline, preoperative levels of sRAGE as compared with their BVD counterparts, then experienced a higher percent and factor increase in sRAGE after CPB. This increase inversely correlated with preoperative SpO_2_ and was positively associated with higher red blood cell transfusion volumes. An understanding of the pathobiology contributing to these sRAGE levels could help shed light on the congenital heart disease patient’s response to CPB.

Patients with SVD had lower preoperative levels of sRAGE when compared to those with BVD. We speculate this difference may be caused by multiple factors including a role for sRAGE as a decoy receptor and the preoperative hemodynamics of a patient undergoing BVD repair. There is a growing body of literature that suggests, while commonly considered a marker of alveolar epithelial injury, sRAGE additionally serves in a protective role in the innate inflammatory response by binding ligands that are destined for RAGE. Interestingly, patients with chronic illness such as diabetes, asthma, and coronary artery disease also have lower levels of circulating sRAGE at baseline, permitting unbound ligands to bind RAGE and perpetuate an inflammatory response through NFkB.^25–27^ Baseline sRAGE levels were lower in our patients with SVD possibly predisposing them to (or caused by) a state of inflammation as seen in these other disease processes.

Another explanation for this finding could lay in the pathobiology of heart failure in the BVD patient prior to surgery. All BVD patients exhibited intracardiac or great vessel-level shunting lesions prior to repair. As pulmonary vascular resistance falls in the newborn period, these lesions result in increased pulmonary blood flow and cardiac volume overload, clinically evidenced in part by pulmonary edema. Preoperative sRAGE levels in BVD patients could be reflective of ongoing pulmonary edema and alveolar injury before a shunting lesion is repaired. Elevated levels of sRAGE have been associated with impaired alveolar fluid clearance, and the utility of trending sRAGE levels in the resolution of lung injury and ARDS is being investigated.^28,29^ Currently, there are no normative data on sRAGE levels in the pediatric population, making it difficult to discern the degree of relative differences in baseline sRAGE levels in SVD and BVD patients.

All patients in our study had higher levels of sRAGE following bypass as compared to their preoperative levels. Liu, et al. found similar results in a study examining sRAGE levels in children undergoing repair for BVD. In this study, however, 16 of the fifty-eight (27.6%) patients developed postoperative acute lung injury as defined by the North American-European consensus criteria.^23,30^ Higher sRAGE levels were further found to be independently associated with severity of lung injury, longer mechanical ventilation time, and hospital length of stay.^23^ As our study population included patients with baseline cyanosis due to their single ventricle physiologies, traditional diagnostic criteria for acute lung injury (alveolar-arterial PaO_2_ gradient, PaO_2_/FiO_2_ ratio, etc.) were not applicable. The median number of days on mechanical ventilation in our population was 0-1 day with hospital length of stay of 6–7 days with no differences between the groups. It should be noted that early extubation was targeted in the postoperative cardiac patient to optimize cardiopulmonary hemodynamics (particularly following Stage II and III palliations) and to limit negative sequelae secondary to mechanical ventilation.^31^

On post-hoc analysis, we found an inverse relationship between preoperative SpO_2_ and sRAGE factor and percent increase after CPB which suggests the degree of cyanosis may be a driving factor for sRAGE protein expression during bypass. Cyanosis is thought to confer an increased susceptibility to both inflammation and reperfusion injury in children undergoing CPB.^32–35^ In a previous study by a member of our group, when patients were divided into normoxemic (>90% SpO_2_) and hypoxemic (</= 90%) groups, myocardial mitochondrial cytochrome c oxidase (CcOx) was found to inversely correlate with the patient’s baseline arterial oxygen saturation, implicating a greater degree of myocardial ischemia-reperfusion injury and potential for increased free radical species generation in patients with lower preoperative oxygen saturation.^35^ Research suggests that cyanosis coupled with hyperoxia employed during CPB may exacerbate the deleterious processes of inflammation and reperfusion injury, both processes in which sRAGE is thought to play a role.

Rong, et al.^36^ demonstrated increased levels of RAGE mRNA and protein expression in the early stages of reperfusion in a canine model of CPB followed by elevated values of downstream markers of inflammation. Further, the group found that a controlled oxygen reperfusion strategy could downregulate RAGE and inflammatory markers, suggesting that post-bypass reperfusion injury and inflammation is in part due to hyperoxia and mediated through RAGE. In a murine model, RAGE null mice survived longer in an induced hyperoxic state and demonstrated less evidence of acute lung injury on histology as compared to their wild-type counterparts.^37^

Hyperoxia during cardiopulmonary bypass has been shown to be neuroprotective as it reduces the load of gaseous micro emboli and offsets the effects of free radical oxidative injury after ischemia reperfusion, and thus, is commonly employed in bypass.^38,39^ Controlled oxygen reperfusion is a different strategy where the FiO_2_ supplied targets an arterial oxygen tension similar to the patient’s preoperative oxygen saturation. This strategy has been shown to downregulate cardiac and systemic markers of inflammation, oxidative stress, and complement activation, notably in patients with SVD as compared to patients with BVD.^33,34,36,38,39^ Investigating the mechanisms by which RAGE participates in these phenomena needs further research.

Lastly, our data show that higher volume of RBC transfusion was independently associated with increased sRAGE levels. This is consistent with an earlier study investigating sRAGE levels after lung transplantation surgery where increased intraoperative RBC transfusion was associated with higher sRAGE levels.^40^ RAGE is detected in low levels in lung endothelium and is inducible when exposed to transfusion of RBCs via a RAGE ligand located on stored RBCs.^41^ It is worth noting that most patients in the SVD group had prior exposures to CPB including Stage I and Stage II operations, and sternal re-entry may have prompted more bleeding, need for transfusion, and chest tube output. Although our center targeted similar transfusion goals for all patients undergoing CPB, transfusion thresholds can vary and are typically higher in patients with SVD compared to BVD counterparts and may motivate the use of more RBC transfusions.^42^ In our study, it is possible that a higher RBC transfusion volume had a causal effect on increasing sRAGE levels to a greater degree in the SVD population, though it is likely other mechanisms play a role. As there is growing concern for increased morbidity due to higher volume RBC transfusion in pediatric cardiothoracic surgery and a shift toward more judicious transfusion strategies, an improved understanding of sRAGE in the congenital heart disease population may pose beneficial.^42–45^ Our study was not designed to evaluate the effect of repeat bypass runs on sRAGE levels; however, sRAGE kinetics have been described in prior CPB studies.^11,22,23^ sRAGE is an early biomarker that increases in concentration rapidly and returns to baseline within 24 hours following CPB. Future studies could investigate the impact of repeat exposures to CPB on sRAGE levels and other markers of inflammation and cellular injury.

There were several limitations to our study. Our sample size was small and heterogeneous, including different morphologic diagnoses, ages, and number of prior operations and interventions. In addition, there is very limited data on normal sRAGE values in the pediatric population making direct comparisons challenging. Our sample collections were at two time points, and longitudinal analysis of sRAGE levels in the postoperative period of SVD patients should be considered in future studies. As mentioned, clinical assessment of lung injury is inherently complicated in the patient with cyanotic congenital heart disease. Long-term follow-up including postoperative hemodynamic assessment and pulmonary function testing may be useful.

In conclusion, children with SVD have greater increases in sRAGE, following exposure to CPB compared to those undergoing repairs of BVD. This may be explained by a unique physiologic response to inflammation, worsening lung injury, hyperoxia and/or exposure to higher RBC transfusion volume. Further research should explore the mechanisms by which sRAGE participates in the perioperative morbidity associated with CPB, as it can potentially be an important novel biomarker for the inflammatory response in children undergoing CHD repair.

## Appendix

### Abbreviations

PaO_2_: Arterial partial pressure of oxygen
AGE: Advanced glycation end products
ARDS: Acute respiratory distress syndrome
BVD: Biventricular heart disease
CPB: Cardiopulmonary bypass
CHD: Congenital heart disease
FFP: Fresh frozen plasma
HMBG1: High mobility group box 1
kg: Kilogram
mL: Milliliter
NFkB: Nuclear factor kappa light chain enhancer of activated B cells
SpO_2_: Peripheral capillary oxygen saturation
pg/mL: Picogram per milliliter
ROS: Reactive oxygen species
RAGE: Receptor for advanced glycation end products
RBC: Red blood cells
SVD: Single ventricle heart disease
sRAGE: Soluble receptor for advanced glycation end products.
